# A de novo complex chromosome rearrangement associated with multisystematic abnormalities, a case report

**DOI:** 10.1186/s13039-017-0332-6

**Published:** 2017-09-02

**Authors:** Chan Tian, Dan Li, Ping Liu, Liping Jiao, Xuefeng Gao, Jie Qiao

**Affiliations:** 1 Center for Reproductive Medicine, Department of Obstetrics and Gynecology, Peking University Third Hospital, Beijing, 100191 China; 2 Key Laboratory of Assisted Reproduction, Ministry of Education and Beijing Key Laboratory of Reproductive Endocrinology and Assisted Reproductive Technology, Beijing, 100191 China

**Keywords:** CCR, SNP-array, FISH, Microdeletion, Rearrangement, AID

## Abstract

**Background:**

Complex chromosomal rearrangements (CCRs) are constitutional structural rearrangements that involve three or more chromosomes or that have more than two breakpoints.

**Case presentation:**

Here, we describe a four-way CCR involving chromosomes 4, 5, 6 and 8. The patient had mild multisystematic abnormalities during his development, including defects in his eyes and teeth, exomphalos and asthenozoospermia. His wife had two spontaneous abortions during the first trimester. The translocations in 4q27, 5q22, 6q22.3, and 8p11.2 were diagnosed by conventional cytogenetic analysis and confirmed by fluorescence in situ hybridization(FISH). After analysis using a SNP array, we defined three microdeletions, including 0.89 Mb on chromosome 4, 5.39 Mb on chromosome 5 and 0.43 Mb on chromosome 8. His mother had a chimera karyotype of 47, XXX[5]/45, X[4]/46, XX[91]; the other chromosomes were normal. After one cycle of in vitro fertility (IVF) treatment followed by preimplantation genetic diagnosis (PGD), they obtained two embryos, but neither was balanced.

**Conclusions:**

The patient’s phenotype resulted from the CCR and microdeletion of chromosomes 4, 5 and 8. The couple decided to use artificial insemination by donor (AID) technology.

## Background

Complex chromosome rearrangements (CCRs) are structural aberrations involving more than two chromosome breaks with exchanges of several chromosomal fragments [1]. The phenotype of individuals with CCRs can be normal; this largely depends on whether or not the CCR is balanced or whether developmentally important gene(s) are disrupted at the breakpoints. Balanced CCRs contain unchanged amounts of genes but unbalanced CCRs do not. According to the literature, approximately 70% of CCRs are detected in people without a phenotype, 20-25% are detected in patients with congenital abnormalities and/or mental retardation and 5-10% are detected during prenatal diagnosis [2]. Occasionally, cases have been detected because of psychiatric trouble [3, 4]. In addition, CCRs are frequently observed in tumor cells, especially in hematological malignancies [5]. Among phenotypically normal CCR carriers, most suffer reproductive failures, including spontaneous abortions, stillbirths, the delivery of children with congenital malformations, and male infertility [6, 7].

There are various classifications of CCRs due to their complex nature. CCRs can be considered familial or de novo, according to the mode of transmission [8]. Based on the number of chromosome breaks, CCRs are divided into two groups: those with four or fewer breaks and those with more than four breaks [9]. CCRs are also divided into three classes according to their structure [10]: 1) three-way rearrangement, which refers to three chromosome breaks and exchanges of chromosomal segments; 2) exceptional CCRs, involving rearrangements in which there is more than one breakpoint per chromosome; and 3) double two-way translocations, which indicates two or three independent, simple reciprocal or Robertsonian translocations that co-exist in the same carrier.

Here, we describe a de novo CCR case that involves four chromosomes and four breakpoints. The patient displayed mild multisystematic abnormalities, which were identified by conventional cytogenetics and molecular genetic technologies. After a failure to obtain normal embryos with PGD, they chose to accept AID with donor spermatozoa.

## Case presentation

A 25-year-old man and his 26-year-old wife were referred to our reproductive medical center due to two spontaneous abortions in the past 3 years after their marriage. The abortions occurred at the sixth and seventh week of gestation for the first and second time, respectively.

**Fig. 1:**
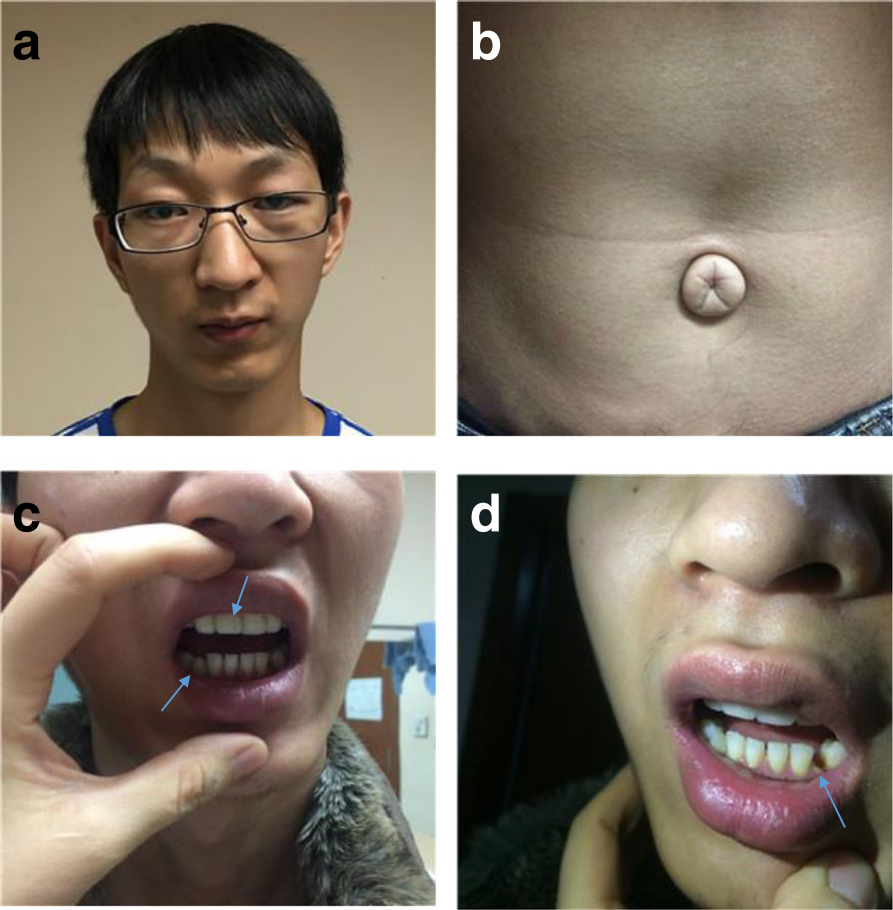
**a** Mug shot of the patient. **b** Exomphalos of the patient. **c** Two central incisors were congenitally lost as implant; the lower right primary canine was retained. **d** The lower left permanent canine was congenitally missing

The physical examination of the husband showed that his eyes had refractive errors; his left eye displayed congenital amblyopia and his vision was 0.2 (Fig. 1a). He also had bilateral primary open angle glaucoma (POAG) and the intraocular pressure of his eyes was more than 40 mmHg. His eyes were subjected to a trabeculotomy and the intraocular pressure was well-controlled. He also had exomphalos (Fig. 1b). His two central incisors were congenitally lost as implant, his lower right primary canine was retained (Fig. 1c) and his lower left permanent canine was congenitally missing (Fig. 1d). He had graduated from high school and is now employed. He can communicate normally. His routine semen analysis demonstrated a sperm deformity rate of 99%, sperm viability rate of 9.56%, DNA fragmentation index (DFI) of 13.58%, and high DNA stainability (HDS) of 15.36%.

He is the second child of non-consanguineous parents and has two sisters. His parents and both sisters are healthy. After discovering his chromosome abnormalities, his family members underwent genetic testing (except his older sister, who was abroad).

After genetic counseling, the couple insisted on preimplantation genetic diagnosis (PGD). Initially, they obtained two embryos to undergo PGD; both were unbalanced. After counseling, they decided to accept artificial insemination with donor spermatozoa.

## Methods and results

Metaphase chromosomes obtained from colchicine-stimulated cultures of peripheral blood lymphocytes and fibroblast cultures were used for GTL-banding and fluorescence in situ hybridization(FISH) analysis. FISH was performed according to the method described by Wieczorek et al. [11]. A SNP-array was performed using Cyto12 genechip (Illumina, USA) according to the manufacturer’s instructions.

**Fig. 2:**
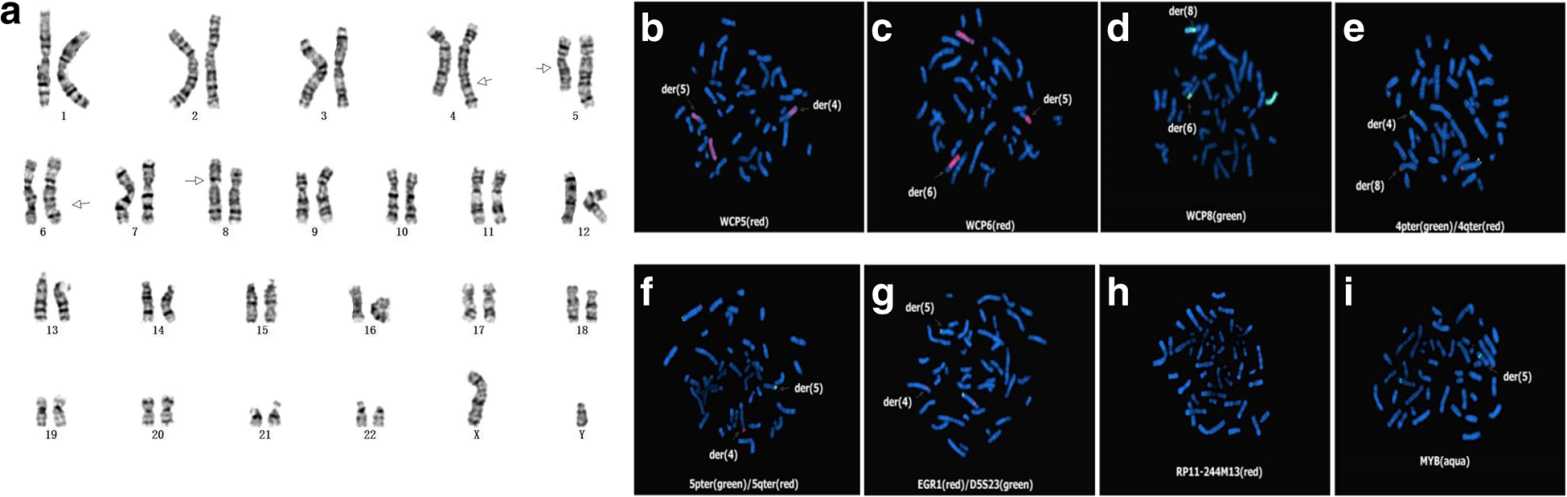
Images of karyotype and FISH. **a**. Metaphase spread in GTG-banding obtained from the patient’s blood lymphocytes showing t(4;8;6;5) (q27;p11.2;q22.3;q22). Arrows show abnormal chromosomes. **b**-**d**. Images of FISH with whole chromosome painting (WCP) probes. Chromosome 5 is *red* in B, chromosome 6 is *red* in C, chromosome 8 is *green* in D. Material from der(4) is present on der(5), whereas material from der(5) is present on der(6). Material from der(6) is present on der(8). **e**-**f**. Images of FISH with terminal probes. 4pter is green, 4qter is red in E. 5pter is green, 5qter is red in F. **g**-**i**. The metaphase spread image of FISH with the EGR (5q31.2) probe in red, D5S23 (5p15.2) probe in green (G), RP11-244 m13(5q21.1q21.2) probe in red (H) and MYB probe (6q23.2) in green (I). [GRCh38/hg38]

The patient, his parents and his younger sister were examined. A classical cytogenetic examination revealed that the patient’s karyotype was 46, XY, t(4; 8; 6; 5) (q27; p11.2; q22.3; q22) (Fig. 2a). The father and younger sister’s G-banded karyotypes were normal. Unexpectedly, his mother’s karyotype was 47, XXX[5]/45, X[4]/46, XX[91] (data not shown). As the patient and his wife had described, his mother was normal during her pregnancy with the patient.

To confirm the presence of a complex translocation involving four chromosomes, FISH was performed with the whole chromosome painting (wcp) probes wcp5, wcp6, wcp8, the chromosome terminal probes 4pter, 4qter, 5pter and 5qter, as well as the specific probes EGR1, D5S23, DMYB and RP11-244 M13. The combined karyotype of the patient was 46, XY,? t(4;8;6;5) (q27;p11.2;q22.3;q22).ish der(4) t(4;8) (q27;p11.2) (4pter+,4qter-,EGR1+,5qter+), der(8) t(8;6) (p11.2;q22.3) (8pter-, 4qter+, WCP8+), der(6) t(6;5) (q22.3;q22) (WCP8+, WCP6+), der(5) del(5) (q21.1q21.3) t(5;4) (q22;q27) (RP11-244 M13-, 5pter+, D5S23+, EGR1-, 5qter-, WCP6+, MYB+) (Fig. 2).

After using wcp, we observed material from der(5) present on der(4), material from der(6) present on der(5), and material from der(8) present on der(6) (Fig. 2b-d). The rearrangement of these chromosomes was confirmed by the terminal and specific probes (Fig. 2e-i). When the probes were hybridized with 4pter and 4qter, we observed that 4qter was present on der(8), whereas 5qter was present on der(4) (Fig. 2e-f). When we used the probes EGR and D5S23, which are specific for 5q31.2 and 5p15.2, respectively, one EGR signal was observed on der(4) (Fig. 2g). When we used the probe RP11-244 M13, which is specific for 5q21.1q21.2, only one copy was observed, indicating a deletion on chromosome 5 (Fig. 2h). A signal from the MYB probe, which is specific for 6q23.2, was observed on der(5) (Fig. 2i). The FISH results were in accordance with the karyotype.

**Fig. 3:**
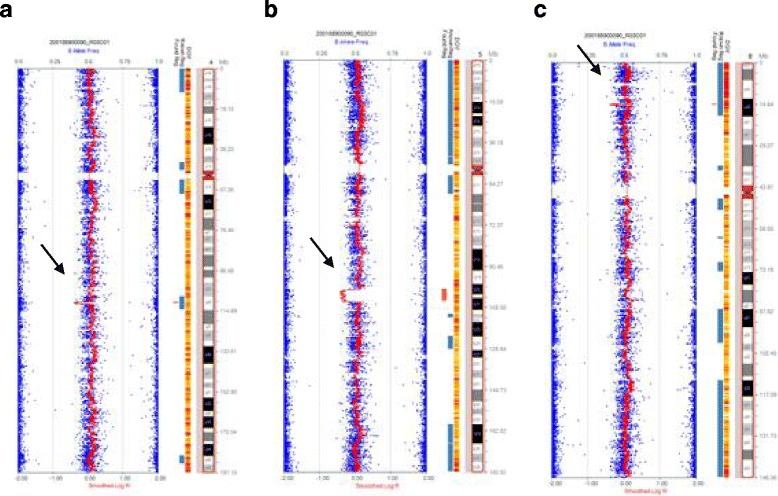
The SNP-array results of the patient. Microdeletions on chromosome 4 (**a**), 5 (**b**) and 8 (**c**) were shown. The arrows indicate the sites of microdeletions

To confirm the FISH results and to determine the presence of microdeletions during chromosome rearrangement, we examined DNA from the patient’s peripheral blood using a SNP-array assay according to the manufacturer’s instructions (Illumina). The results were as follows: arr[hg19] 4q25(110499958-111,393,691)×1, arr[hg19] 5q21.1(100718992-106,104,465)×1 and arr[hg19] 8p22(14547284-14,972,402)×1, which revealed three microdeletions on three different chromosomes (Fig. 3). The genes involved in these regions are shown in Table 1. These genes included five OMIM genes (which belong to the phospholipase A2 group XIIA (PLA2G12A)), ELOVL fatty acid elongase 6 (ELOVL6), solute carrier organic anion transporter family member 4C1 (SLCO4C1), diphosphoinositol pentakisphosphate kinase 2 (PPIP5K2, also known as HISPPD1) and nudix hydrolase 12 (Nudt12). These genes are not associated with known disorders. Among them, SLCO4C1 is an organic anion transporter, HISPPD1 is a kinase (which acts as a cell signaling molecule), and the remaining genes are enzyme-encoding genes that are involved in several metabolic processes, including phospholipid, fatty acid and nucleotide metabolism. Interestingly, two genes, RRH and LRIT3, were related to his ocular disorder. RRH (retinal pigment epithelium-derived rhodopsin homolog) belongs to the seven-exon subfamily of mammalian opsin genes [12]; mutation of this gene has been linked to retinitis pigmentosa and allied diseases [13].

**Table 1:** Detailed information on the microdeletions resulting from chromosomal rearrangement

Chromosome	Genomic position (GRCh37/hg19)	Cytobands	Size (M kb)	Copy number	Genes
4	110,499,958-111,393,691	q25	0.89	1	CCDC109B; CASP6; PLA2G12A; CFI; GAR1; RRH; LRIT3; EGF; ELOVL6;
5	100,718,992-106,104,465	q21.1 q21.2 q21.3	5.39	1	SLCO4C1; SLCO6A1; PAM; GIN1; HISPPD1; C5orf30; NUDT12; RAB9P1;
8	14,547,284-14,972,402	p22	0.43	1	SGCZ; MIR383;

The LRIT3 (leucine rich repeat, Ig-like and transmembrane domains 3) encoded protein may regulate fibroblast growth factor receptors and affect the modification of these receptors, which are glycosylated differently in the Golgi and endoplasmic reticulum. Mutations in this gene are associated with congenital stationary night blindness, type 1F [14]. Our results demonstrate that although these genes are not associated with known disorders, they show haploinsufficiency.

**Fig. 4:**
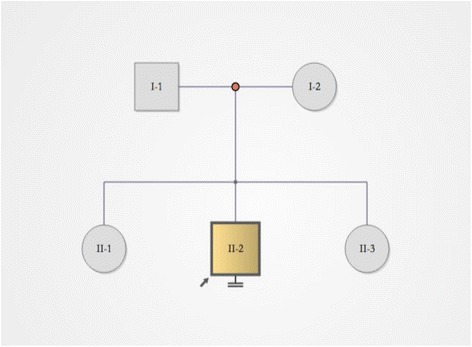
The CCR patient’s pedigree. I represents the patient’s parents; II represents the patient and his sisters; II-2 is the patient

To determine whether the patient’s microdeletions were inherited from his parents or whether they appeared de novo, the parents were subjected to a SNP-array analysis. The results demonstrated that the parents do not have microdeletions in the three chromosomes mentioned above (Fig. 4), indicating that the loss of chromosome fragments was derived from rearrangement.

## Discussion and conclusion

Here, we describe a four-way CCR involving several microdeletions on chromosomes 4, 5, 6 and 8. The patient had mild multisystematic abnormalities during development, including defects in his eyes and teeth, exomphalos and asthenozoospermia. After completing a cycle of PGD, he did not obtain normal embryos and decided to use AID.

CCRs are rare events with an estimated frequency of 0.1% [15]. Most CCR cases are unknown to the carriers or their families. Some chromosomes, including 2, 3, 4, 7, and 11, are more frequently implicated in CCR than would be expected. This is the first CCR case to involve chromosomes 4, 5, 6, and 8 [16, 17].

CCRs can involve up to 15 breakpoints. According to a 2011 summary, cases that included four breakpoints accounted for 29.1% of all 251 CCR cases [2].

However, this CCR occurred de novo; the patient’s mother’s karyotype was 47, XXX[5]/45, X[4]/46,XX[91], and she had a low level of mosaic 47, XXX and 45, X, which was less than 10%. She had three children at 19, 21 and 35 years of age and had no fertility issues during her childbearing age. Her mosaic karyotype is possibly due to a gain or a loss of X chromosomes as she aged [18, 19] or chromosomal nondisjunction during the culture of peripheral blood lymphocytes.

Breakpoint analysis of a growing number of complex rearrangements has revealed that translocations involving three or more chromosomes are likely formed via chromothripsis [20–23]. Most constitutional chromothripsis events occur de novo and those investigated thus far have been verified as paternal in origin [20–23]. Alternatively, mitotic errors in the early embryo [24] or the pulverization of micronuclei [25] could be responsible for numerous DNA breaks. We speculate that this de novo CCR is due to chromothripsis.

According to the literature, a three-way CCR would theoretically form 64 different gametes: one normal, one balanced, and the rest unbalanced [2]. A four-way CCR, as in this case study, has a probability of producing normal and balanced gametes of less than 1/32. We disclosed this possible risk and as a result of genetic counseling, the couple opted for PGD. After a failure to obtain normal embryos with PGD, they chose to accept AID with donor spermatozoa.

In conclusion, we systematically investigated this CCR and the accompanying microdeletions and were able to characterize the genetic defects that resulted in the patient’s multisystematic abnormalities, which had bothered him for many years. After receiving genetic counseling, the couple understood that they could not conceive a chromosomally balanced child because the husband had microdeletions in three chromosomes. They chose to undergo AID.

## Abbreviations

AID: Artificial insemination by donor
CCR: Complex chromosome rearrangements
FISH: Fluorescence in situ hybridization
PGD: Preimplantation diagnosis
